# Harnessing Multi-Omics Strategies and Bioinformatics Innovations for Advancing Soybean Improvement: A Comprehensive Review

**DOI:** 10.3390/plants13192714

**Published:** 2024-09-28

**Authors:** Siwar Haidar, Julia Hooker, Simon Lackey, Mohamad Elian, Nathalie Puchacz, Krzysztof Szczyglowski, Frédéric Marsolais, Ashkan Golshani, Elroy R. Cober, Bahram Samanfar

**Affiliations:** 1 Agriculture and Agri-Food Canada, Ottawa Research and Development Centre, Ottawa, ON K1A 0C6, Canada; siwar.haidar@agr.gc.ca (S.H.);; 2 Department of Biology, Ottawa Institute of Systems Biology, Carleton University, Ottawa, ON K1S 5B6, Canada; 3 Agriculture and Agri-Food Canada, London Research and Development Centre, London, ON N5V 4T3, Canada

**Keywords:** soybean, multi-omics, integration, genomics, transcriptomics, proteomics, metabolomics, epigenomics, phenomics

## Abstract

Soybean improvement has entered a new era with the advent of multi-omics strategies and bioinformatics innovations, enabling more precise and efficient breeding practices. This comprehensive review examines the application of multi-omics approaches in soybean—encompassing genomics, transcriptomics, proteomics, metabolomics, epigenomics, and phenomics. We first explore pre-breeding and genomic selection as tools that have laid the groundwork for advanced trait improvement. Subsequently, we dig into the specific contributions of each -omics field, highlighting how bioinformatics tools and resources have facilitated the generation and integration of multifaceted data. The review emphasizes the power of integrating multi-omics datasets to elucidate complex traits and drive the development of superior soybean cultivars. Emerging trends, including novel computational techniques and high-throughput technologies, are discussed in the context of their potential to revolutionize soybean breeding. Finally, we address the challenges associated with multi-omics integration and propose future directions to overcome these hurdles, aiming to accelerate the pace of soybean improvement. This review serves as a crucial resource for researchers and breeders seeking to leverage multi-omics strategies for enhanced soybean productivity and resilience.

## 1. Introduction

Soybean (*Glycine max* (L.) Merr.) is a highly important agricultural crop worldwide with vast implications in human diet, animal feed, and sustainable agricultural practices. Through a symbiotic relationship with soil bacteria, soybean has the capacity to fix inert atmospheric nitrogen into more biologically available nitrogen compounds. Incorporating soybeans into crop rotations reduces the need for nitrogen fertilizers, which minimizes the release of nitrogen compounds as environmental pollutants. With high seed oil and protein, soybean is used in a wide range of consumer-end products and animal feed. With some of the highest seed protein content among legumes, soybean is one of the most widely consumed crops in the world due to its dense nutritional value. In fact, much evidence suggests that expansion of soybean production is an excellent avenue to address the caloric and protein requirements of a growing population [1].

Soybean research supports efforts to navigate changing and unpredictable climates, as well as emerging and spreading agricultural pests and disease, and the needs of a growing population. One area of study is driving soybean agriculture to more northern locations in Canada, China, and the United States [2]. With widespread global interest in soybean agriculture, a suite of ever-growing resources for soybean improvement and breeding strategies has become invaluable to the soybean research community.

Historical improvement of the soybean has relied largely on the use of classical genetics, phenotypic selection, and the pedigree method. Large segregating populations are grown and observed for phenotypic variation from which individuals that display traits conferring desirable agronomic and seed quality characteristics are selected to continue in subsequent generations, until segregation is limited and most traits are considered fixed, usually in four or five generations. At this point, and once adequate seed is available, cultivars can be bulked for testing at a larger scale, where agronomic performance, especially yield, as well as resistance to abiotic and biotic stressors, can be assessed in multiple environments. Only the highest performers are selected for release to growers, representing a tiny fraction of the original segregating population. This process, from the original hybridization to cultivar release, can take up to 10 years or more, unless it is accelerated by costly greenhouse generational advancement or winter nurseries in the opposite hemisphere.

Multi-omics refers to the integrated analysis of data from multiple “omics” disciplines to obtain a comprehensive view of biological processes. In recent years, multi-omics approaches coupled with bioinformatics tools have emerged as powerful strategies for enhancing soybean improvement efforts [3]. These integrated methodologies offer a comprehensive understanding of the complex molecular networks underlying various traits of interest in soybean, including yield, seed quality, and abiotic and biotic stress tolerance [4]. By simultaneously analyzing multiple layers of biological information such as genomics, transcriptomics, proteomics, metabolomics, epigenomics, and phenomics, researchers can unravel the complex interactions between genes, proteins, and metabolites, thereby providing valuable insights into the mechanisms governing soybean traits [5].

While the application of multi-omics approaches in soybean research has accumulated significant attention, similar efforts have been undertaken in other important crops, including maize [6], rice [7], and wheat [8] as well as fruit crops like tomato [9]. Recent reviews have highlighted the utility of multi-omics techniques in elucidating the genetic and molecular basis of key agronomic traits in these crops, paving the way for targeted breeding strategies [10,11,12]. These reviews underscore the broad applicability of multi-omics approaches across diverse plant species and emphasize their potential to revolutionize agricultural practices worldwide.

In light of these biotechnological advancements, the objective here is to delve into the latest developments in multi-omics techniques and bioinformatics applications specifically for soybean research. By reviewing recent studies and discussing the potential implications for soybean breeding programs, we aim to provide insights into how multi-omics approaches can contribute to the accelerated development of agronomically enhanced soybean varieties. Through a comprehensive exploration of an array of different omics approaches and powerful data processing tools we provide a review on the current state of multi-omics and bioinformatics in soybean biology.

## 2. Pre-Breeding and Genomic Selection

Efforts must be made early in the pipeline to streamline the process and guarantee a greater chance of success. One way plant breeders are tackling this long timeline is to carry out pre-breeding or germplasm development. Pre-breeding is the process of introgressing desirable alleles from a source with characteristics that often make it unsuitable for commercial release into a background that can then be used as a suitable donor into more commercially acceptable cultivars [13]. The initial cross between the exotic donor and the intermediary is never intended for commercial release; rather, it acts as a stepping stone from which the trait can be transitioned into multiple backgrounds depending on the other traits the breeder is trying to capture [14]. By developing these intermediaries in advance, breeders are ensuring that a larger proportion of the early generation material will be suitable for selection, reducing the time and effort required between the cross and the cultivars release.

Genomic selection employs and builds on the key aspects of phenotypic selection while adding genotypic information to assist the breeder in selecting high-performing individuals [15]. The basics of this selection method are fairly straightforward: populations are divided into a training set and a testing (breeding) set. Both sets are genotyped, after which only the training set is grown and phenotyped. The resulting phenotypic data are then used in conjunction with the genotypic data to develop the genomic selection model, to be used in predicting the phenotypic traits of the testing set, called genomic estimated breeding values. The breeder then selects lines observed and/or predicted to be the highest performers. Importantly, genomic selection has been shown to improve genetic gain in breeding populations while reducing loss of potentially important genetic diversity [16].

Marker-assisted selection (MAS) is an important tool contributing to the success of both pre-breeding and genomic selection strategies [17]. MAS allows breeders to selectively screen parental lines for one or a few desirable alleles, making choices at the outset that guarantee a larger number of desirable offspring regardless of selection method employed. Several techniques exist for MAS, such as CAPS/dCAPS, SSR, and SNP based KASP [18]. Cleaved amplified polymorphic sequencing (CAPS) and derived cleaved amplified sequencing (dCAPS) rely on gene-specific DNA amplification with or without inclusion of the target SNP in the primer sequence, which then can produce diagnostic bands after restriction enzyme digestion of varying size on an agarose gel depending on the alleles present [19,20]. Simple sequence repeat (SSR), sometimes referred to as microsatellite markers, is another PCR-based approach in which varying numbers of repeated sequences are diagnostic for the allele present, with results again read from a gel [21]. The Kompetitive Allele Specific PCR (KASP) assay is diagnostic for the SNP present in a DNA sample, with primers specific to the gene and allele suspected to be present in the sample. KASP is significantly less ambiguous than CAPS/dCAPS and SSR markers and does not require any sort of gel electrophoresis to make genotype calls. KASP is seen as a more modern technique for MAS due to its scalability, repeatability, and specificity [22].

## 3. Multi-Omics Approaches and Bioinformatics Tools and Resources in Soybean Research

### 3.1. Genomics

Genomics is the study of genes within an organism and the ways those genes function and interact, as well as how those genes behave, function, and interact under different environmental conditions [23]. Cells within an organism, like soybean, for example, all contain a complete copy of the organism’s genome, but carry out a vast array of functions and interactions that allow the organism to thrive in the environment for which it is adapted. Studying and understanding the genomics of an organism helps to explain basic biological functions, and allows for improved understanding of how genetics is able to fine-tune and control the organism’s phenotype [24].

The study of soybean genomics has the potential to greatly improve our understanding of soybean biology, especially as it relates to agronomic performance and farmgate profitability. The soybean genome is an ancient paleopolyploid, first sequenced by Schmutz et al. [25], revealing 1.1 giga base pairs with a predicted 46,000 protein coding regions. More recently, reference grade genome assemblies have been reported by Song et al. [26] and Valliyodan [27], who chose to improve on the originally published reference assembly of the Williams 82 cultivar using updated techniques and technologies. Furthermore, Valliyodan et al. [27] reported a reference grade assembly of the Southern US variety Lee, Shen et al. [28] published a complete genome of the Chinese cultivar Zhonghuang 13, and Xie et al. [29] reported a reference genome of *Glycine soja* W05, a representative of the soybean’s wild crop relative. Having quality reference genomes, with the ability to choose the one most appropriate for the study being carried out, is fundamental to the advancement of the field of soybean genomics.

Reference grade genomes are essential, but the assembly of pan-genomes allows for a more critical look at the genomics of the soybean, and the differences between groups of individuals and their conserved and dispensable genomic regions [30]. The concept of pan-genome was intended to better reveal and describe the totality of the genome of a species, rather than the genome of a single individual. Several key pan-genomes have been assembled and reported for the soybean. Torkamaneh [31] assembled a pan-genome of only cultivated soybean, from diverse geographies and genetic backgrounds, revealing that more than 90% of the genome is conserved and demonstrating that the genomic regions available for genetic gains in adapted cultivars is highly restricted. Bayer et al. [32] and Liu et al. [33] assembled pan-genomes that included a greater diversity of individuals. One approach, used by Bayer et al. [32], collected over 1100 genome sequences to assemble the pan-genome, showing that significant loss of allelic diversity occurred during domestication, and that only a small number of founders has contributed to most of the genetic variability employed in today’s commercial cultivars. In contrast, Liu et al. [33] employed phylogenetic analysis to select 26 individuals for a pan-genome, capturing wild, landrace, and commercial cultivars. The three studies outlined above all agree that the soybean has a highly conserved genome between individuals, that genetic bottlenecks of domestication represent a major loss of potentially economically important alleles, and that pan-genomes should be employed to focus efforts on specific genomic regions that are highly variable for crop improvement provided that they determine useful trait variation.

Next-Generation Sequencing (NGS) methods have allowed for a vast increase in the amount of genomic data available for soybean research [34]. This increase in available data heightens the demand for tools that can process and annotate extensive amounts of information more quickly [35]. New bioinformatics tools have been developed to fill this processing need. The Extensive De novo Transposable elements Annotator (EDTA), for example, is a comprehensive pipeline used to simplify the process of creating a transposable element library for annotation [36]. This was accomplished by benchmarking many of the most commonly used programs using various metrics and incorporating the best performing options into the pipeline [36]. Bioinformatics tools are also used to identify proteins and sequences responsible for certain regulatory functions. iTAK is a computational program designed to identify transcription factors (TF), transcription regulators (TR), and protein kinases (PK) from a given sequence. This was achieved by comparing TF/TR classification rules in various databases and deriving a consensus of rules that can be applied based on existing literature [37]. The identification of structural rearrangements (inversion, duplications) and mutations (SNP, Indels) is also vital for understanding gene function [38]. The Synteny and Rearrangement Identifier (SyRI) tool was developed to identify these variations and structural rearrangements within all syntenic and structurally rearranged regions of related genomes [39]. Other tools such as MCScanX [40] and i-ADHoRe [41] also provide a means for synteny detection, visualization, and comparison.

Pinpointing SNPs associated with phenotypic changes in a genome as large as the soybean’s requires the use of large and diverse collections of individuals along with mathematical and statistical techniques. Genome-wide association studies (GWAS) use a large panel of individuals that represent the diversity employed by plant breeders in their crop improvement efforts, as well as wild crop relatives and historical landraces [42,43]. GWAS denotes SNPs that are significantly associated with a phenotype and have been widely used to suggest genomic regions and hotspots. The diversity of phenotypes studied using GWAS points toward its acceptance in the field of genomics and its applicability for crop improvement: resistance to soybean mosaic virus [44], primary root length [45], photosynthesis [46], protein content [47], flowering time [48], mineral element uptake [49], stem pushing and lodging resistance [50], and tolerance to cold imbibition stress [51], to name a few.

Unlike GWAS, genomic selection is an applied genomics technique that feeds directly into the plant breeding pipeline by reducing the effort required for variety development and release. While this technique is being applied in increasing frequency, studies reporting its use are still somewhat limited. Miller et al. [52] used large breeding populations tested in multiple environments with relatively low genotyping density, revealing that things like population relatedness, marker density, and genomic models are all factors in the success or failure of the technique. Kaler et al. [53] compared the ratio of training to testing set populations, finding that it was more important to choose more significant SNPs in genotyping rather than altering the ratio of training to testing sets. Bandillo et al. [54] assessed whether genomic selection was useful in improving yield, using seven years of yield data to show that it was just as effective as standard breeding at phenotypic selection while reducing the overall number of lines needing to be grown each year.

Genomics provide a detailed understanding of the genetic architecture and functional mechanisms of soybeans. The advancements in soybean genomics, from the initial sequencing to the development of reference-grade assemblies and pan-genomes, have significantly enhanced our knowledge of soybean biology and its potential for crop improvement. Techniques such as NGS generate the data used in approaches like GWAS and genomic selection, which are crucial for identifying key genetic variations, understanding complex trait inheritance, and improving breeding efficiency. As research progresses, the integration of advanced bioinformatics tools with multi-omics approaches will continue to help contribute to sustainable agricultural practices and food security.

### 3.2. Transcriptomics

Development of high-throughput sequencing technologies has brought about a new era of molecular biology. No longer constrained to the confines of real-time quantitative PCR (RT-qPCR) and Sanger sequencing, high-throughput sequencing has released a flood of information that provides a snapshot of a sample under any given condition. This snapshot of transcript information allows researchers a peek into the ongoing biology in a sample at any given time. In combination with whole genome sequence assembly, reference genomes support transcriptomic analysis, offering a universal reference for researchers to compare gene expression data. However, the value of transcriptomics extends beyond reference genomes; RNA sequencing (RNA-seq) reads can be assembled de novo should a reference genome be unavailable or inappropriate for the given study. This circumvents the challenge of using microarray chips, which require known sequence information for chip development.

Transcriptomics opens a world of possibilities for pairwise comparative studies, known as differential expression (DE) analysis. Tools such as DESeq2 [55] and edgeR [56] are useful for calculating DE from RNA-seq data. DE analysis quantifies the relative expression levels of active genes between two groups, such as different tissue types of one sample, identical samples under different treatments, or different samples under the same treatment, among other possible comparisons.

RNA-seq data can be entirely vast, but purposeful in the right hands. One RNA-seq dataset can be used to investigate the data holistically, or be sliced into genes/gene families of interest for a targeted approach. The development of user-friendly RNA-seq atlases puts the power of RNA-seq in the hands of any user, bypassing any requirement for plant growth and wet-lab work (assuming the data are suitable for the research question). This means that a near-infinite number of RNA-seq and DE studies can be investigated at a minimal cost. Users can download RNA-seq data to carry out pairwise DE analyses tailored to their own research interests.

Severin et al. [5] developed a tissue-specific and temporally variable RNA-seq database, publicly available through SoyBase (https://soybase.org/soyseq/ (accessed on 24 September 2024) [5,57]). The RNA-seq atlas allows soybean researchers to search candidate genes and receive corresponding transcriptomic data across all tissues and stages of development. More recently, Almeida-Silva et al. [58] developed the Soybean Expression Atlas v2, a publicly available RNA-seq database spanning transcript- (measures the expression of individual RNA variants) and gene-level expression data (combines the expression of all variants of a gene) for 5481 soybean samples [58].

Liu et al. [59] integrated single-cell RNA-seq (scRNA-seq) and spatial transcriptomics to produce a cell atlas across multiple histological features of a soybean root nodule when inoculated with rhizobia [59]. The cell atlas allows the researchers to identify expression profiles of cell types within different parts of the root and nodule and use this information to identify rare cell subtypes that play key roles in maturation and function of the nodule. This study also uncovered functionally distinct and transitional cellular subgrouping between infected and uninfected cells. This cell atlas is publicly available (https://zhailab.bio.sustech.edu.cn/single_cell_soybean (accessed on 24 September 2024), [59]) to facilitate soybean root and nodule research through cell type-specific transcriptomics.

Further, RNA-seq data and phenotype data can be computationally combined using Weighted Gene Co-expression Network Analysis (WGCNA), a novel analysis technique that groups samples by phenotype and uses the corresponding RNA-seq data to identify co-expressed gene networks that differ between phenotypic groups. Typical network analyses assess co-expressed genes regardless of phenotypic trait status; WGCNA differs from trait network analysis by sub-setting the expression data by phenotypic groups to look for gene networks that differ between groups, which is important for identifying trait-associated gene networks [60]. WGCNA has been used in soybean pathology; in a recent study on plant–pathogen interaction between *G. max* and soybean mosaic virus, hub genes and key gene regulatory pathways involved in pathogen response were identified [61]. WGCNA has also been used to identify genetic factors that have implications on embryo size and hormone signaling pathways [62]. More recently, an advanced multi-WGCNA package has been released, which allowed for trait-specific WGCNA using more than one phenotypic trait [63], which has valuable uses in complex transcriptomic experiments.

In a bottom-up or targeted approach, gene family analysis can be conducted using RNA-seq data, providing direct information on the transcriptional activity of a family of sequentially or functionally related genes across all given samples. As an example, an investigation into the *Glycine max* Sugars Will Eventually be Exported Transporter (*GmSWEET*) family was carried out to expand on the recently characterized seed protein QTL identified as *gmsweet39* [64] to be positively correlated with seed oil content [65]. Using transcriptomic analysis of 10 soybean lines over four years, this study assessed expression variability across all known *GmSWEET* genes between soybeans grown in two geographically distinct regions of Canada [66]. Interestingly, a difference in *gmsweet29* and paralog *gmsweet34* expression pointed to a putative link to seed protein content differences observed between locations [66]. In a study on the environmental effects on seed protein in soybean, large-scale transcriptomics was used for pathway mapping of DE genes involved in the alanine–aspartate–glutamate metabolism pathway. This study identified an important difference in expression of genes underlying asparagine metabolism in soybeans grown in cooler, drier climates, and this likely influences seed protein content [67,68].

In another study, Lopes-Caitar et al. [69] conducted an in silico investigation of the *Hsp20* genes in soybean in response to temperature and nematode infection. A combination of multiple soybean expression databanks were then used to obtain expression data in silico across all the identified *Hsp20* genes, including the SoyBase RNA-seq Atlas [5]. In addition to identification of groups of *Hsp20* genes induced under temperature stresses (hot and cold), this study identified a conserved promoter sequence unique to nematode stress response-*Hsp20* genes [69].

Undoubtedly, transcriptomics has had a profound impact on accelerating soybean research, among many other fields of research, due to its widespread application. Here we provide an overview of novel uses of transcriptomics in soybean research, paving an ever-expanding path for the usage of RNA-seq tools and techniques.

### 3.3. Proteomics

Proteins are the foundation of biological processes and are fundamental in understanding soybean biology. Proteomics offers a detailed evaluation of all the proteins that are present at a particular time. The evolution of proteomics (large-scale study of proteins, particularly their structures, functions, and interactions within a biological system) has greatly aided soybean research, especially regarding growth, stress responses, and nodule development and functioning [4]. Soybean proteome studies initially depended on 2D gel electrophoresis, prior to the revolutionary impact of liquid chromatography coupled with tandem mass spectrometry (LC–MS/MS) on high-throughput proteomics, which improved both efficiency and accuracy [70]. Studies by Hajduch et al. [71] constructed high-resolution proteome maps of soybean seed filling, which set a guide for subsequent proteomics advancements [71].

Subsequent studies, like Afroz et al. [72], have explored tissue-specific analyses in soybean seedlings, revealing key proteins in various organs such as leaves, hypocotyls, and roots [72]. Analyses of root hairs and developing seeds using isobaric tags for relative and absolute quantitation (iTRAQ) have identified specific proteins associated with root hair and seed development [73,74]. Proteomics has also been employed to study enzyme expression and the regulatory mechanisms involved in their accumulation during seed storage [75]. For instance, Xu et al. [76] used proteomics to compare protein expression between high-oil and high-protein soybean cultivars, shedding light on differences in oil synthesis [76]. It was found that when *GmDGAT1-2* was overexpressed, there was a significant alteration of total fatty acid content through an upregulation of oleosin and a downregulation of the enzyme lipoxygenase [77].

Proteomics has revealed critical proteins involved in stress responses. Studies like those by Xu et al. [77] and Wang et al. [78,79] identified tissue-specific abiotic stress responses in soybean, highlighting the mechanisms at play. The studies utilized advanced proteomic techniques such as LC-MS/MS to identify and analyze the proteins involved in stress responses in soybean. In 2017, Wang et al. [79] conducted proteomic analyses focused on specific tissues of soybean seedlings exposed to flooding condition [79]. In another study, they documented the stress responses of young plants and seedlings exposed to combined stresses in a tissue-specific manner and identified sensitive tissues based on protein profiles during different developmental stages [80]. In 2021, using quantitative proteomics, Wang et al. [78] conveyed the effects of calcium on soybean radicle protrusion during germination using LC-MS/MS for proteomic analysis to identify and quantify protein expression changes in response to varying calcium levels. Their results highlighted that low levels of calcium promoted radicle protrusion, whereas higher levels of calcium suppressed it [78]. Aside from abiotic stress responses, Yadav and Singh [81] discuss how soybean plants change their protein expression in response to infestation by *Spodoptera litura* (a common cutworm) [81]. Using LC-MS, they found that 390 differentially abundant proteins are involved in the response to infestation. These proteins are involved in secondary metabolism, reactive oxygen homeostasis, and signaling pathways and could be possible candidates for further analysis [81].

Moreover, recent quantitative proteomic analyses have revealed insights into soybean seed traits under various stressors, paving the way for targeted breeding efforts. Islam et al. [82] carried out a quantitative proteomic analysis comparing low-linolenic-acid transgenic soybean seeds silenced for the *GmFAD3* fatty acid desaturase gene with control seeds. They found disruptions in proteins involved in fatty acid metabolic pathways, noting a decreased abundance of proteins related to fatty acid initiation, elongation, desaturation, and the β-oxidation of α-linolenic acids [82]. Wei et al. [83] studied the impact of temperature and humidity stress on the vigor of soybean cotyledons, embryos, leaves, and pods using a combination of quantitative proteomics with physiological data [83].

Given that proteins are responsible for many cellular functions, there is a lot of unique information that can be gleaned from understanding a plant’s proteome [84]. Bioinformatics plays a vital role in developing novel analysis strategies for the ever-increasing amount of proteomics data [85]. Data-independent acquisition (DIA) is an emerging mass-spectrometry-based technique for collecting proteomics data. However, identifying peptides directly from DIA is difficult as the data are often highly multiplexed. PECAN is a free library tool developed to accurately detect peptides directly from DIA data based on ion product scoring [86]. Bioinformaticians have also developed platforms dedicated to bridging the gap between informatics and biological researchers. For example, Perseus is a user-friendly, computational platform dedicated to the analysis of proteomic data. This platform provides data including protein expression, post-translational modifications, interaction proteomics, and time-series analysis [87]. Traditionally, protein quantification is primarily achieved using isotope-based labeling methods. The label preparation requires several preparation steps; it is therefore of interest to develop methods to quantify proteins without prior labeling. MaxLFQ was developed as a label-free protein quantification tool. It was implemented into the MaxQuant platform and uses a novel protein identification approach to maximize the ratio information from peptide signals, thus increasing the accuracy of the quantification [88].

While soybean proteome research lags behind other crops, it serves as a foundational platform for functional genomics studies. Advancing soybean research could be achieved through the development of a reference map for the soybean proteome [4]. Proteomics data can aid in identifying new proteins, analyzing the expression patterns of their corresponding genes, and facilitating their molecular cloning. The integration of proteomics with other -omics data holds promise for identifying elite alleles and developing molecular markers, crucial for soybean molecular breeding. Despite challenges in proteomics, analytical techniques continue to progress, offering greater insights into protein-specific influences on cellular processes. Nevertheless, understanding the molecular mechanisms underlying soybean traits requires efficient, high-throughput techniques for analyzing protein expression and interactions, driving soybean research into new areas of research [89].

### 3.4. Metabolomics

Metabolomics is an emerging field within systems biology, and it offers a novel approach to analyzing small metabolites in plant tissues [90]. Techniques employed for quantifying plant metabolites include thin-layer chromatography (TLC), liquid chromatography–electrochemistry–mass spectrometry (LC–EC–MS), gas/liquid chromatography–mass spectrometry (GC/LC–MS), nuclear magnetic resonance (NMR) spectroscopy, Fourier transform infrared (FT–IR) spectroscopy, direct infusion mass spectrometry (DIMS), and capillary electrophoresis–LC–MS; with LC–MS, GC–MS, NMR, and capillary electrophoresis MS being the most prevalent in plant studies [12,91]. These techniques allow for direct links between metabolites and observable traits, as metabolites can affect both gene transcription and protein expression [92]. By revealing metabolic fingerprints through metabolite detection and bioinformatics analysis, researchers gain insights into plant metabolism, shedding light on crucial pathways like the tricarboxylic acid cycle and glycolysis [93]. Additionally, single-cell mass spectrometry offers insights into cellular physiology and hidden phenotypes by allowing the analysis of gene expression, protein function, and metabolite levels in individual cells [94]. Its application, although still evolving, holds potential for deciphering cellular mechanisms in both biomedical and environmental contexts [95].

Researchers have examined how seed dry weight, seed coat color, and maturity impact metabolite levels. For example, studies on black soybean seeds have explored how metabolite levels vary at different maturity stages, revealing that many metabolites change as seeds mature and that isoflavone content is closely linked to seed maturity [96]. Furthermore, by manipulating specific metabolic pathways, it was possible to increase the nutritional value of genetically modified soybeans by boosting isoflavone accumulation in developing seeds [96,97]. Metabolomics also aids in understanding how soybeans respond to abiotic stress, highlighting the role of certain metabolites, like glycine and proline, as osmoprotectants [98]. Moreover, it identifies metabolic markers indicative of drought stress, offering potential applications in crop improvement and breeding by providing insights into the metabolites involved, thus providing new opportunities to increase crop yields [98].

In soybean, metabolomics has revealed 169 metabolites crucial for seed development, including those involved in amino acid biosynthesis, antioxidant utilization, and lipid oxidation [99]. Additionally, there were notable differences in metabolite levels and distinct correlations between metabolites across various soybean cultivars under different shade/light conditions. The isoflavone profiles of soybean germplasm underscored the wide range of isoflavones found in soybeans [100], and various aglycones were linked to differing degrees of shade tolerance in seedlings [101]. Moreover, an NMR-based approach to observe the influence of silver nanoparticles on the metabolites of transgenic soybean varieties was performed, and it was conveyed that silver application does not play a role in secondary metabolites [102]. In a targeted metabolomics approach, Nguyen et al. (2024) revealed the metabolomes of 64 seed lines of *G. soja* [103]. In a more untargeted metabolomics approach, the differences between high- and low-production poly(γ-glutamic acid) was observed, and it was found that among thousands of differentially expressed metabolites, 257 were significantly either upregulated or downregulated [104].

Despite these advancements, challenges persist in metabolomics, including the complexity of plant metabolic pathways and the need for integration with other approaches [105]. While metabolomics has accelerated our understanding of plant metabolism and genetic architecture, gaps remain between research findings and practical applications. Efforts to resolve methodological issues and integrate metabolomics with other disciplines hold promise for future breakthroughs [4].

### 3.5. Epigenomics

Epigenetics refers to heritable changes in gene expression that do not alter the underlying genetic sequence. Chromatin conformation and DNA accessibility are modulated through histone modifications, and DNA markers such as methylation and acetylation. The collection of epigenetic variations across the genome is referred to as the epigenome, and the soybean epigenome is a unique landscape due to its paleopolyploid history [25,106,107]. The major contribution of epigenomic studies to understand soybean biology has been the comprehensive mappings of the epigenetic repatterning within its genomic landscape. Studies have consistently found that not only do differentially methylated regions exist within the soybean genome, but these regions also correlate with differential expression patterns. Pericentromeric and centromeric genomic regions are found hypermethylated at a higher rate than non-pericentromeric regions, and they have lower gene densities and decreased transcription levels [107,108,109,110]. A significant portion of the soybean genome is composed of hypomethylated regions termed DNA methylation valleys (DMVs) and has been found to be enriched with transcription factor genes [109]. Transposable elements are preferentially methylated in all three contexts (pericentromeric, centromeric, and non-pericentromeric regions) compared to protein coding genes and, consequently, have decreased expression levels [107,108,109]. The evolutionary development of the intrinsically linked genome and epigenome has dictated the genomic architecture and dynamic regulatory responses that modern soybean uses to modulate its phenotypic responses, providing the plants with the ability to grow, adapt, and survive.

Epigenetic regulation is implicated in the facilitation of various physiological processes in soybean, with dynamic changes in marker levels documented during seed maturation, germination, root nodulation, and flowering [108,109,111,112,113]. Using whole-genome bisulfite sequencing (WGBS), it has been observed that DNA methylation levels change significantly in cotyledons as they mature, with transcribed genes approximately two-fold more likely to be differentially methylated than non-transcribed genes [108]. In addition to DNA methylation, fluctuations in histone modifications appear to be an integral part of the gene regulatory network underlying seed maturation. Chromatin immunoprecipitation (ChIP) assays found genes located in DMVs enriched in histone marks on several lysine residues at all seed and germination stages, with no significant enrichment of the histone marks found at any developmental stage [109]. Importantly, the proportion of histone marks on DMV genes varied throughout development and in parallel with gene expression levels.

Modulation of epigenetic marks have also been shown to play a role in soybean’s response to various stressors [114,115,116,117,118,119,120,121,122,123,124,125,126,127,128,129]. Near-inbred lines (NILs) derived from resistant and susceptible soybean lines were found to have significant differences in their methylome profiles, both before and following infection with soybean cyst nematode (SCN) [124]. In response to SCN infection, both NILs exhibited an induction of genome-wide DNA hypermethylation of miRNA genes, but with susceptible lines having a much higher occurrence of differentially methylated miRNAs. Consistent trends have also been observed between differential N^6^-methyladenosine (m^6^A) modifications and the pattern of transcriptional responses initiated by the soybean when exposed to stressors such as *Meloidogyne incognita* infection or heavy metals such as lead or cadmium; all showed dynamic redistribution of m^6^A modifications across transcripts, and generally genes with m^6^A modification had higher expression levels than those without this modification [118,119,129]. Conjoint analysis of differentially methylated genes (DMGs) with DEGs in each of these studies provided candidates that may play important roles in modulating these responses. In addition to helping plants overcome environmental perturbations, soybeans pre-exposed or “primed” to stressors have shown more prompt responses, long-term increased resistance, and decreased functional consequences, as well as epigenetic memory (the ability to pass favorable phenotypic responses to their progeny) [125,128,130,131,132].

An epigenetic regulatory mechanism for conferring salt tolerance in soybean has been elucidated, whereby salt stress induces the expression and accumulation of a nuclear factor Y subunit, which directly interacts to destabilize and promotes histone acetylation and subsequent activation of salt-responsive genes to enhance salt tolerance [122]. Liu et al. [133] identified and molecularly characterized six soybean *LSD-like* genes, *GmLDLs*, which were found to exhibit catalytic demethylase activity, elucidating their potential function in acting as chromatin remodeling factors of stress-inducible genes to regulate expression in response to different abiotic stresses.

Importantly, while studies focusing on epigenomic mapping have been immensely beneficial to furthering our knowledge regarding soybean biology, there remains significant challenges in solidifying causal relationships due to the complex interplay between environmental influences, genetic, and epigenetic factors. Moving forward, more emphasis should be placed on the testing of hypothetical causal relationships between identified epigenetic marks and their suspected functional consequences, which can only be facilitated through the integration of a multi-omics approach and the implementation of epigenome editing tools. More researchers are utilizing an integrative approach to comprehensively analyze and construct the complex regulatory networks controlling phenotypic responses in soybean [113,123,124,131,132,134].

### 3.6. Phenomics

Since the development of high-throughput phenotyping technologies, research in the area of crop phenotyping is now being acknowledged as ‘phenomics’ [135]. Manual study of plant phenotypes is inefficient and costly on a large scale and prone to errors due to human bias. In order to address these challenges, plant phenomics is playing a key role in helping to improve efficiency and accuracy in studying plant characteristics [136], and it has been observed that the study of phenomics has increased in recent years [4].

Soybean cultivars differ based on many factors, including but not limited to resistance to stress and metabolic capacity, which influences resulting seed attributes like size and composition. Significant changes in structural characteristics can arise from even the most minor developmental differences [137]. Morrison et al. (2021) used 3D depth cameras to measure canopy height in soybean and compared it to single point systems operated by hand, and found that there were significant differences [138]. Plant phenomics uses advanced, high-throughput phenotyping technologies to gather detailed phenotype data throughout plant growth. This approach generates extensive trait data, allowing for the division of a single trait into several smaller, testable components [139]. Using fixed, quantitative, and uniform standards for data collection enables automated, high-throughput analysis [140,141]. This improves the precision of crop phenotype identification and boosts the efficiency of plant breeding and cultivation management [141,142].

Industrial unmanned aerial vehicles (UAVs) equipped with advanced high-definition dual-camera multispectral systems can accurately estimate soybean yields and classify pod maturity by analyzing time-series multispectral images [143]. Integration of high-spatial-resolution red green blue (RGB), multispectral, and thermal data from UAVs has enhanced the accuracy of assessing soybean physiological and biochemical parameters, such as chlorophyll content and nitrogen concentration, as well as biophysical traits like leaf area index and biomass [144]. Additionally, early-season RGB images of the soybean canopy have been used to predict yield, maturity, and seed size based on color and texture features [145].

These UAVs, outfitted with costly multispectral, hyperspectral, and thermal imaging equipment, are increasingly utilized for precise sampling of soybean canopy traits, including height, area, temperature, and leaf wilting [146,147,148]. Despite early success studying several phenotypes, obtaining high-dimensional phenotypic traits from 2D images remains challenging, with some morphological trait estimates requiring calibration. To address this challenge, 3D reconstruction of plant morphology from 2D images using open-source structure from motion (OpenSfM) and multi-view stereo (MVS) methods has been employed [135]. This approach allows for the generation of high-density 3D point clouds to capture plant height and growth phenotype data [149]. A notable study used 3D reconstruction technology to analyze the phenotypic fingerprint and growth patterns of soybean plants throughout their development, demonstrating a cost-effective alternative to expensive laser scanners and potential for automating certain processes [150].

High-throughput phenotyping methods can allow for effective assessment of plant responses to stressors and subsequently the identification of novel genes [151]. For instance, in studying stress-induced effects on plants, a combination of approaches, namely ground vehicles, unmanned aerial systems, and smartphone-captured digital images, can be employed to routinely and directly evaluate the severity of iron deficiency chlorosis (IDC) in real time, thereby enabling large-scale field screening for soybean IDC tolerance [152,153]. Zhou et al. (2018) demonstrated the feasibility of an automated plant phenotyping system in a greenhouse setting, using digital cameras to continuously capture images and extract features such as chlorophyll content and salt tolerance, showcasing its potential for analysis [154]. Additionally, for stressors like flooding, Zhou et al. (2021) applied multispectral and infrared thermal imaging cameras to take canopy images from varying flight altitudes, employing deep learning techniques to estimate soybean flooding damage scores, thus facilitating the identification of genetic features linked to abiotic stress and the development of resilient soybean varieties [147]. Plant phenomics research is experiencing rapid growth due to advancements in remote sensing, robotics, visualization, and artificial intelligence (AI), which present unprecedented challenges in managing large datasets and numerous images. Machine learning (ML), particularly deep learning, is being employed to address these challenges, using techniques such as convolutional neural networks (CNNs), multilayer perceptrons (MLPs), and recurrent neural networks (RNNs) [155,156]. CNNs, in particular, have found widespread application in plant phenotyping tasks, supported by advances in cloud and graphics processing unit (GPU) computing [155].

In soybean yield prediction, ML, multimodal data fusion, and deep learning techniques have been applied to UAV-collected data with notable success [157,158,159]. A fusion architecture that utilizes multi-view images has been developed to monitor soybean pods and estimate yield, demonstrating effectiveness [160]. Similarly, a novel image analysis method has been devised to rapidly assess the morphology and color of soybean seeds [161]. Algorithms such as random forests [162] and deep CNNs are well suited for vision-based tasks, enabling the detection of materials in harvested soybeans on a large scale [163]. Furthermore, for below-ground root phenotype data, a fully automated pipeline employing deep learning architectures has significantly reduced labor and inconsistency in nodule detection and segmentation (i.e., separation of the image into regions with similar features), allowing for earlier assessment of genetic and environmental impacts on nodules [164].

While soybean phenomics research has seen significant progress, its focus has predominantly been on describing external physical traits of plants, neglecting internal and biochemical characteristics. This limitation has hindered its practical application in plant breeding. However, private breeders are increasingly utilizing advanced technologies, such as drones, to gather comprehensive phenotypic data. To fully leverage the potential of plant phenomics in advancing plant breeding, it is crucial to integrate the study of internal physiological traits and biochemical characteristics alongside external traits.

## 4. Integration of Multi-Omics Data for Soybean Trait Improvement

Combining multi-omics data—genomics, transcriptomics, proteomics, metabolomics, epigenomics, and phenomics—is essential for a comprehensive understanding of biological processes in soybean. Each omics layer provides unique insights into the different aspects of cell function and regulation. Genomics reveals the genetic blueprint, while transcriptomics highlights active gene expression. Proteomics uncovers the proteins and their interactions, metabolomics identifies metabolic pathways and their products, epigenomics sheds light on gene regulation mechanisms beyond the DNA sequence, and phenomics links these molecular data to observable traits [4,10,11,91,136]. Integrating these datasets allows for a holistic view of how genes and their products interact within the complex biological networks of soybean.

A multi-omics approach is especially helpful in understanding stress responses and adaptation mechanisms in soybean; by integrating these data, candidate genes and regulatory networks that contribute to key agronomic traits can be identified and harnessed for strategic breeding. For example, under drought conditions, changes in gene expression and metabolite levels were correlated to identify key pathways that confer drought tolerance [165]. This integrated approach enables the identification of biomarkers for breeding and genetic engineering, which can improve soybean varieties for enhanced stress tolerance and yield [98,165].

One notable example of multi-omics integration in soybean research is the identification of novel genetic markers associated with soybean seed weight and oil content. By combining genomics and transcriptomics, researchers identified QTLs and candidate genes that influence both seed weight and oil content [166]. Kumar et al. (2021) used an integrated approach combining genomics, transcriptomics, and proteomics to identify molecular mechanisms behind seed oil and protein content [167]. Another example is the study of soybean resistance to biotic stress, specifically resistance to the soybean cyst nematode (SCN), a major pest affecting soybean yields [168,169,170]. An integrated approach combining transcriptomics with metabolomics was used to identify resistance genes and understand their expression patterns in resistance to SCN, where significantly different metabolites involved in specific pathways related to sensitive or resistant varieties were revealed [169]. Moreover, a study employed ML to integrate genomic data from GWAS and phenotypic data from hyperspectral reflectance, identifying genetic loci linked to yield, and this allowed the development of predictive models with improved accuracy [159].

Additionally, multi-omics integration was used to study soybean responses to phosphorus deficiency, a common nutrient stress. Transcriptomics analysis showed how the genes identified were associated with phosphorus uptake and utilization, and how they were differentially expressed under phosphorus stress [171]. Proteomics and metabolomics analyses identified proteins and metabolites involved in phosphorus metabolism. This integrated approach led to the identification of key genetic and biochemical pathways involved in phosphorus use efficiency, offering targets for improving soybean nutrient management [171].

Multi-omics can be especially useful when used together to characterize novel whole genome sequences and subsequently investigate gene expression to gain a comprehensive appreciation for genetically important and/or active areas of the genome. Gupta et al. (2023) used DNA sequencing to assemble the complete genomes of three different isolates of soybean rust pathogen (*Phakopsora pachyrhizi*) collected from different areas of South America [172]. This study investigated the evolutionary history and the influence of the high transposable element content found in the *P. pachyrhizi* genomes. These researchers used raw RNA-seq data to determine heterozygosity and perform SNP analysis across the three different exomes [172]. SNP analysis was used to point toward unique nonsynonymous mutations for each fungal isolate, and transcriptomics was used to determine expression activity of each of the corresponding genes [172]. This study used a combination of omics techniques to draw meaningful conclusions on the natural history of this large and detrimental fungal family, and the implications on adaptation and genetic plasticity as a result of high transposable elements within their genomes. This is also an excellent example of using RNA-seq data beyond the usual DE analysis.

CRISPR (clustered regularly interspaced short palindromic repeats) technology has significantly enhanced the integration of multi-omics techniques by providing a precise and efficient method for genome editing. For example, CRISPR has been used to knock out or modify genes in soybean, enabling researchers to observe changes in gene expression, protein levels, and metabolic pathways resulting from these edits [173,174,175]. These targeted modifications help in understanding the functional roles of specific genes and their contributions to complex traits such as plant architecture and flowering time.

The use of CRISPR also facilitates the validation of candidate genes identified through multi-omics integration. By editing these genes, researchers can confirm their roles in specific biological processes and pathways. For instance, CRISPR has been used to validate genes involved in soybean’s response to abiotic stresses such as drought and salinity [176]. This approach not only accelerates the identification and validation of key genetic markers but also aids in the development of improved soybean varieties with desired traits. Thus, CRISPR technology plays a crucial role in advancing multi-omics research by enabling precise genetic manipulations and facilitating the comprehensive study of gene function and regulation [177].

This technology allows researchers to introduce specific genetic modifications and study their effects across various -omics layers [178,179]. Wan et al. (2022) used CRISPR/Cas9 to mutate the *E1* early maturity locus in soybean to confirm its functional role in plant development. Phenotypic evidence identified the role of *E1* on initiation of terminal flowering, determinate growth habit, and branch number. Transcriptomics and DE analysis between the wildtype (WT) and mutant pointed to a compensatory response in the expression of *E1* homologs, *E1-La* and *E1-Lb*. Expression profiles of WT and mutant plants identified that the mutants had a significant decrease in *Dt1* expression and significant upregulation of *Dt2* expression, both growth habit regulator genes [180,181], suggesting that *E1* regulates growth habit using the *Dt2-Dt1* signaling pathway [182]. This research combines the power of CRISPR/Cas9 gene editing with transcriptional profiling to identify a key regulatory mechanism with important agronomic implications.

Large-scale multi-omics methods are an excellent approach to identifying the etiology of a gene or trait. Liang et al. (2022) used GWAS to identify a locus on chromosome 18, *Dt2*, with a predominant association to shoot branching in soybean, an agronomically valuable trait that influences yield [183]. Using transcriptomics, the authors pointed to a difference in expression of *Dt2* corresponding with natural variations in sequence between major haplotypes. Expression data from 20 random natural accessions identified a negative correlation between *Dt2* expression and branch number [184]. The authors used CRISPR/Cas9 to knock out *Dt2* and observed that the mutant had an increased number of branches, delayed flowering and maturity, with increased main stem node number and plant height [184], consistent with previously reported functions [181,185]. The authors used DE to identify the interaction between *Dt2* and the promoter region of *GmAp1* gene family representatives. This study exhibits a seamless flow from genomics, to transcriptomics, to functional validation of the underlying genetic basis of an agronomic trait.

Bioinformatics tools have been developed for each of the -omics disciplines and have helped in data analysis and processing. However, in order to gain a holistic understanding of soybeans, these platforms need to be integrated and examined together [186]. Yang et al. developed SoyMD, a multi-omics database that allows for the querying of information regarding a gene of interest including functional annotation, homology, genetic variation, and epigenetic signals. SoyMD integrates several -omics tools to achieve the highest level of detail for its assemblies [187]. This includes annotations using Gene Ontology (GO), RNA-seq data that was aligned using HiSat2, and epigenetic signals that were predicted using Basenji, a deep CNN approach [188,189,190]. SoyMD is implemented as a web application and provides an invaluable tool for multi-omics analysis of soybean [187]. SoyOmics is another integrated multi-omics publicly available database that aims to gain a more holistic understanding of soybean. SoyOmics gathers assembled genomes, RNA-seq data, and DNA methylation datasets. These data were used to create toolkits such as easy GWAS for quick-start GWAS analysis, and ExpPattern for expression pattern analysis. SoyOmics is available as a web application and provides additional resources for multi-omics analyses for soybeans [191]. There are also various bioinformatics tools that can be used to interact with these multi-omics platforms. Pathway tools (PTools) interact with the Pathway Genome database (PGDB) and allow for genome analysis, metabolic modeling, and analysis of high-throughput data from this database. This includes data querying and data visualization, which allows for researchers to easily obtain their data of interest as quickly as possible [192].

A comprehensive understanding using an integrated multi-omics approach is crucial for developing strategies to optimize soybean growth and productivity under diverse environmental challenges, ultimately contributing to food security and sustainable agriculture. Table 1 presents a summary table of the main findings discussed in this section as well as their references.

## 5. Novel Approaches and Emerging Trends

Emerging techniques in soybean -omics are revolutionizing our understanding of this essential crop. scRNA-seq, as discussed above and in Liu et al. (2023), is emerging as a powerful tool to study gene expression at the individual cell level, offering unique resolution in understanding cellular heterogeneity and developmental processes in soybeans [168]. Often, it is difficult to distinguish between technical artifacts in scRNA-seq data, as generated by cells with a lot of noise, which results in a distinct gene expression pattern from actual biological heterogeneity between cells. The Single-cell RNA-seq Quality Control (SinQC) tool was developed and uses the cells of the main population as controls to establish quality cutoff points, which allows it to identify these technical artifacts [193]. Another promising technique is GWAS, which leverages large datasets to identify genetic variations linked to important agronomic traits [194]. These techniques, combined with advanced bioinformatics tools, are enabling researchers to dissect complex genetic traits and accelerate the development of superior soybean varieties [179].

The integration of novel -omics techniques holds great potential for accelerating soybean improvement efforts and overcoming longstanding agricultural challenges. High-throughput sequencing and GWAS enable the identification of genetic markers associated with desirable traits, which can be utilized in MAS to expedite the breeding process [51]. This accelerates the development of soybean varieties that are better adapted to diverse environmental conditions. By integrating these advanced techniques, researchers can tackle existing challenges in soybean cultivation, leading to increased productivity and sustainability.

Less common than GWAS, genome-wide epistatic studies (GWES) use similar concepts of diverse panels of individuals along with mathematical and statistical techniques, but it points toward pairs of SNPs or markers that in combination are statistically associated with significant phenotypic differences [195]. GWAS excels at detecting single polymorphisms in the genome associated with the phenotype but does not consider that highly repetitive genomes like the soybean may have multiple mutations working in conjunction to control phenotypic differences [195,196]. GWES can act as a stepping stone between soybean genomics and the genomics of model organisms as it can reveal more complex gene networks at play that are not readily observable and can point out upstream or downstream functional information that contributes to the field of functional genomics more readily than the results of a simple GWAS study. Moellers et al. [196] used GWES to explore resistance of soybean to *Sclerotinia sclerotiorum*, demonstrating that even though single loci with high effect are elusive, multiple loci working in collaboration can provide important levels of resistance while at the same time shining light on the immune system of the soybean. Assefa et al. [197] used GWES to explore -IDC- in soybean, showing that GWES also has applicability in studying abiotic stressors. The limitations of GWES lie in its computational cost, which is significantly higher than GWAS, and also the need to functionally validate two loci working in conjunction rather than a single locus alone.

Analysis of latent phenotypes (i.e., nuance variations pervasive in regulatory circuits), together with GWES represent cutting-edge approaches that fill critical gaps in current soybean research and breeding strategies. Latent phenotype analysis involves the use of advanced imaging or computational techniques to uncover hidden traits that are not easily observable through traditional phenotyping methods [198]. This approach can reveal subtle variations in plant morphology, physiology, and development, providing a more comprehensive understanding of trait penetrance and genetic diversity in soybean populations [199]. By identifying these latent phenotypes, traits that may contribute to improved yield, stress tolerance, and overall plant performance can be selected. By incorporating latent phenotype analysis and GWES into soybean research, scientists can gain a deeper understanding of the genetic basis of complex traits and develop more effective breeding programs to enhance soybean productivity and resilience [199].

Machine learning and AI are revolutionizing soybean research and breeding by providing advanced analytical tools to manage and interpret large datasets. These technologies enable the identification of data and the modeling of complex relationships that are too difficult to accomplish with traditional methods [200]. Machine learning methods, such as the random forest classifier, have been used to identify important phenotypic predictor variables related to seed yield. This information can then be used by breeders to improve breeding selection, thus optimizing the selection and placement in agro-management systems [201]. AI has also seen a surge in application to multi-omics analysis [202]. More specifically, deep learning, a subset of ML, has been used to develop pDeep, a neural-network based model that can predict the intensity distribution of product ion mass spectrometry based proteomics analysis to predict fragmentation spectra for any peptide using only its sequence [203]. Deep learning has also been used in the development of Deepnovo, a neural network model that can perform de novo peptide sequencing using mass spectrometry data without the use of an existing database [204]. AI is also seeing expanded uses in genomics, where deep learning was used to develop DeepBind, a software tool used to predict the sequence specificity of DNA and RNA binding proteins with high accuracy [205]. Deep learning was also used to develop DeepSEA, another tool that can predict chromatin effects of sequence alterations with single nucleotide precision [206]. AI and ML are also used to optimize existing processes. CRISTAI uses an ML-based algorithm to help with sgRNA design for use in CRISPR-Cas9 genome editing. The CRISTA algorithm has been shown to have higher accuracy than traditional methods for predicting the propensity of a genomic site to be cleaved by the designed sgRNA [207].

## 6. Challenges and Future Directions

While multi-omics investigations have near endless potential for advanced bioinformatic exploration, challenges in integrating information across multiple platforms can negatively influence results by introducing ambiguity, reduced confidence, or miscommunication across platforms, to name a few examples.

Updated reference genomes, such as the movement in the soybean research community from Williams 82.a2.v1 to Williams 82.a4.v1 and/or Williams 82.a6.v1, change the standardized baseline (genome) for which a vast number of -omics studies depend. While updates to the reference genomes are crucial to the advancement of soybean research, discrepancies between older and newer reference genomes introduce limitations in the cross-talk between data generated across different reference genomes. This can lead to inaccurate gene and/or marker mapping, hindering any potential for contributing to the improvement of soybean breeding programs.

Large scale -omics quickly generate a vast amount of data, which poses issues regarding optimal storage and organization (both long and short term). Furthermore, bioinformatic algorithms may not necessarily be equipped or suitable beyond a particular size threshold, and scalability needs to be considered; further, this threshold may vary across -omics platforms. Tools and databases are being developed at a rate that is overwhelming for many researchers. It can therefore be difficult to ensure that all these new tools adhere to findable, accessible, interoperable, reusable (FAIR) research standards [208]. Additionally, multi-omics approaches often result in larger datasets than many researchers are used to working with. In order to maximize the potential of these approaches, a higher emphasis on interpretation of large datasets, coupled with a strong understanding of the biological systems being studied, will need to be applied [11]. There are also persisting challenges with each of the individual -omics techniques that hinder the multi-omics integration. For example, RNA-seq data is often generated using complex plant tissues, which results in noise from transcripts that are outside of the region of interest. Single-cell RNA-seq offers a potential solution to this noise by removing the noise from the unwanted cells, but it is cost-prohibitive in many cases at this time [209].

Large-scale multi-platform data also pose a challenge in interpretation of the results. Interpretation of multi-omics results needs to be conducted within the biological context of the data. Tissue type, developmental stage, sample type (DNA, mRNA, protein, metabolite, etc.), and experimental condition must be carefully coordinated and interpreted such that the biological context of the information is not lost. Data visualization across multi-omics platforms poses an issue regarding graphically presenting information taken from multiple contexts (i.e., transcriptomics and metabolomics—the transcriptional activity and the metabolite distribution can easily be displayed alone, but how can these data effectively be shown within the same graphical context?).

While adopting a multi-omics approach to study the intricacies of biological systems is an attractive option due to the significant amount of data generated from multiple layers of study, it is not without significant challenges. Not only does each -omics method generate substantial volumes of data, but the data generated from different -omics techniques each have their own structures and scales, resulting in highly dimensional data. This presents computational challenges in data handling operations, in which we are restricted by algorithm and tool development, processing power, and storage capacity. Additionally, as an emerging field, the integration of multi-omics studies currently has no standardization regarding experimental protocols, data collection, data processing and statistical modeling, presentation, and result dissemination. Lack of standardization in experimental conditions, data collection, and data processing methods leads to issues in the reproducibility of results and application of findings to other experimental conditions. The large amounts of highly dimensional and heterogenous data create statistical challenges, with large numbers of non-independent variables increasing noise and risk of false positives, tendency of overfitting, and multicollinearity in statistical models, resulting in decreased power and difficulty establishing thresholds and cutoffs. While statistical significance does not always indicate biological relevance, the lack of accurate statistical models subsequently makes interpreting the results from complex interactions between -omics layers more difficult and hinders strategies for candidate selection for further functional validation. Importantly, even with the progress being made within this sphere, we continue to remain limited by the functional annotation of genes, proteins, and metabolites, with a tendency of bias toward well-annotated genes remaining strong. The future of multi-omics investigative studies carries astronomical potential for gaining a holistic understanding of biological activity from multiple perspectives at the same time. More work is needed to advance the seamless flow from one -omics platform to another. As the field of bioinformatics progresses, new and enhanced algorithms will be developed, which will lead to more robust bioinformatic analyses—for example, a platform where the effects of genetic or epigenetic modifications can be pre-tested in silico before experimental verification. This would revolutionize the approach to plant research, allowing for precise predictions and optimizations.

Recent technological advancements in throughput, multiplexing, resolution, and accuracy now allow for the simultaneous profiling of multiple -omes from single cells with unprecedented clarity and sensitivity, providing a comprehensive and holistic view of complex cellular processes. The advent and application of AI-assisted techniques, such as machine learning and deep learning, to biological analyses have improved, and continue to improve, our ability to overcome the limitations posed by traditional analysis methods. Integration of multi-omics studies with advanced AI learning models will provide a framework for efficiently identifying patterns, extracting meaningful relationships from extensive datasets, interpreting complex and intricate data layers, and ultimately accelerating multiple facets of plant research. Further, hardware will also advance as we look toward the future of -omics and agriculture; for example, new UAVs and drones will be equipped with cutting-edge cameras, sensors, and information relay signaling. Multi-omics analysis has opened an entirely new world of analytics in the fields of molecular biology, microbiology, agronomy, and so much more. Moreover, addressing these advancements will require a focus on education, including the need for training at university levels to equip the next generation of scientists and researchers with the necessary skills to meet these challenges.

Emphasis now needs to be placed on creating more intuitive bioinformatics pipelines that are capable of multi-omics analysis. Pipelines such as Genpipes and MOMIC represent a promising beginning as they are both recently developed pipelines that perform multi-omics analyses [210,211]. By complementing these pipelines with additional tools for data visualization, and potentially including graphical user interfaces in new pipelines, which can improve accessibility, multi-omics analysis could reach new heights of applicability.

## 7. Conclusions

The integration of multi-omics strategies with bioinformatics innovations is a transformative approach in soybean improvement. These techniques collectively offer a multidimensional, holistic view of soybean biology, encompassing the genetic blueprint (genomics), gene expression patterns (transcriptomics), protein functions and interactions (proteomics and interactomes), metabolic pathways (metabolomics), epigenetic modifications (epigenomics), and observable traits (phenomics). By integrating these diverse data layers, researchers can unravel the complex regulatory networks and molecular mechanisms driving soybean growth, development, and responses to environmental stresses.

Such comprehensive insights enable precision breeding strategies that are more targeted and efficient. By pinpointing specific genes and pathways associated with desirable traits, breeders can develop soybean varieties with improved yield, quality, and resilience. This is especially crucial in the context of climate change, where crops must adapt to increasingly variable and extreme conditions. Enhanced stress tolerance in soybeans, for instance, can lead to varieties that maintain productivity under drought, heat, or pest pressures.

Moreover, the integration of multi-omics data with bioinformatics tools accelerates the discovery and functional validation of candidate genes, reducing the time and resources required for developing new soybean varieties. This innovation also supports sustainable agricultural practices by facilitating the creation of crops that require fewer inputs like fertilizers and pesticides, thereby minimizing environmental impact. Advanced bioinformatics tools can sift through vast amounts of data to identify patterns and correlations that might not be apparent through traditional methods, making it possible to predict how different genetic variations will affect the plant’s phenotype. This predictive power is crucial for breeding programs aiming to produce robust soybean varieties capable of thriving under various environmental conditions.

The multi-omics approach also aids in understanding the epigenetic modifications that regulate gene expression in response to environmental factors. These modifications can have profound effects on plant traits and can be harnessed to develop soybeans with enhanced adaptability and performance. By leveraging epigenomics, researchers can identify epigenetic markers associated with desirable traits and use this information to select parent plants for breeding programs or influence the plant performance in situ in a temporary manner [212].

Furthermore, phenomics, which involves the comprehensive measurement of phenotypes on a large scale, enables the detailed assessment of how genetic variations manifest in observable traits. High-throughput phenotyping allows for the rapid and accurate collection of data on plant characteristics, facilitating the identification of phenotypic traits linked to specific genetic and epigenetic variations. This integration of phenomics data with other omics data provides a complete picture of the plant’s biology, guiding breeders in their efforts to enhance soybean varieties.

Ultimately, the continued advancement and application of multi-omics and bioinformatics in soybean research are vital for ensuring global food security and sustainability. Collaboration among scientists, breeders, and bioinformaticians is essential to fully exploit these technologies’ potentials, leading to strong and resilient soybean varieties that can meet the growing demands of the world’s population while preserving environmental health. This collaborative effort will drive innovation in agricultural practices, paving the way for a future where soybeans and other crops are optimized for both productivity and sustainability.

## Figures and Tables

**Table 1:** A summary of the main findings and the reference, as well as which -omics technique was used.

Main Finding	Genomics	Transcriptomics	Proteomics	Metabolomics	Epigenomics	Phenomics	Reference
Key pathways that confer drought tolerance		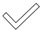		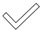			[165]
Novel QTLs and candidate genes that influence both seed weight and oil content	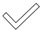	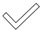					[166]
Molecular mechanisms behind seed oil and protein content	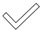	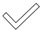	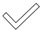				[167]
Resistance genes and understanding their expression patterns in resistance to SCN		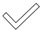		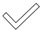			[168,169,170]
Genetic loci linked to yield	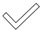					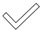	[159]
Key genetic and biochemical pathways involved in phosphorus use efficiency		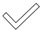	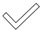	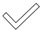			[171]
Natural history and implications on adaptation and genetic plasticity of *Phakopsora pachyrhizi*	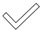	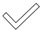					[172]
*GmSPL9* genes in different combinations alter plant architecture in soybean	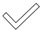	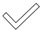				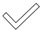	[173]
Roles of phytochromes in regulating photomorphogenesis and flowering time	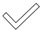	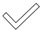				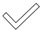	[175]
Validation of genes involved in soybean’s response to abiotic stresses such as drought and salinity	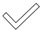	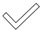				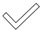	[176]
*E1* early maturity locus in soybean regulates stem growth using the *Dt2-Dt1* signaling pathway	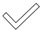	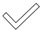				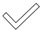	[180,181,182]
Interaction between *Dt2* and the promoter region of *GmAp1* gene family representatives	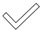	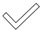					[183,184]
SoyMD, a multi-omics database for information regarding a gene of interest including functional annotation, homology, genetic variation, and epigenetic signals	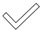	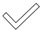			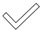	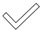	[187]
SoyOmics, an integrated multi-omics publicly available database that aims to gain a more holistic understanding of soybean	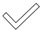	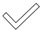			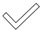		[191]
Pathway tools (PTools) allow for genome analysis, metabolic modeling, and analysis of high-throughput data from this database	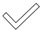	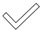		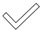			[192]
